# Cervical cytology and the diagnosis of cervical cancer in older women

**DOI:** 10.1177/0969141315598174

**Published:** 2015-09-07

**Authors:** Rebecca Landy, Alejandra Castanon, Nick Dudding, Anita Wey Wey Lim, Antony Hollingworth, Willie Hamilton, Peter D Sasieni

**Affiliations:** 1 Queen Mary University of London, Charterhouse Square, London, UK; 2 Sheffield Teaching Hospitals NHS Foundation Trust, Sheffield, UK; 3 Whipps Cross University Hospital, Barts Health NHS Trust, London, UK; 4 College House, St Luke's Campus, Exeter, UK

**Keywords:** Cervical cancer, cervical cytology, positive predictive value, sensitivity, specificity, pap test, early diagnosis, survival

## Abstract

**Objectives:**

Most non-screen-detected cervical cancers are advanced stage. We assess the potential for cytology to expedite diagnosis when used outside of routine call and recall screening for cervical cancer.

**Methods:**

Two cohorts of women with cytology that did not appear to have been taken as part of routine screening, nested within a census of cervical cytology, in England between April 2007 and March 2010 were studied: 93,322 women aged 40–69 at first cytology, and 14,668 women aged ≥70. The diagnostic performance of high grade cervical squamous intraepithelial lesion (HSIL) or worse cytology was estimated. We also estimated case-fatality from stage distribution in women aged ≥66 with and without cytology in the year prior to diagnosis.

**Results:**

There were 259 cancers diagnosed in women aged 40–69 at first cytology, and 78 in women aged ≥70. The sensitivity of cytology ≥ HSIL for cancer was 89% and 83% respectively, and the number of women needed to test to identify one cancer was 404 (95% confidence interval [CI]: 355–462) and 226 (95% CI: 177–292) respectively. Women aged ≥66 with cytology within a year of diagnosis had earlier stage cancers than those without, corresponding to a 17–22% reduction in case fatality.

**Conclusions:**

Cervical cytology is an excellent identifier of cancer among women tested outside routine screening call and recall. Its use as a triage tool, for instance in women with vague gynaecological symptoms, could facilitate earlier stage diagnosis and reduce cervical cancer mortality.

## Introduction

Global strategies for cervical cancer control include vaccination against high risk HPV types, population screening, and early-stage diagnosis.^1^ While the first two are currently given appropriate consideration, strategies for down-staging non screen-detected cervical cancer have been overlooked. Cervical cancer in those who are too young to benefit from screening, who are no longer offered screening, or who have not been screened recently is often diagnosed at advanced stage and has poor prognosis.

The aim of cervical screening is to prevent cervical cancer, through identifying and treating pre-invasive cervical lesions. Additionally, screening can lead to the early diagnosis of cervical cancer, though that is not its main focus, and this has not been explored. In England, it has been shown that one cancer is diagnosed for every 2726 women screened,^2^ and that the sensitivity of screening cytology (even at a cut-off of high-grade cytology) to invasive cancer is high.

This paper considers the use of cervical cytology as an aid to diagnosis of invasive cancer, with the aim of down-staging cancers, outside of routine call and recall for cervical screening. We show the distribution of cytology results (both in all women with cytology and in those with cervical cancer) for two groups of women in whom cytology was unlikely to have been conducted for screening alone; women whose first cytology test was carried out aged 40–69, and women with cytology aged ≥70 (ie. no longer offered cervical screening). We also compare the stage distribution in women beyond the age of last screening invitation with and without cytology in the year prior to diagnosis, and estimate the difference in case fatality.

## Methods

We studied a retrospective cohort of women in the general population who had cervical cytology taken in the 12 months prior to a diagnosis of cervical cancer, between April 2007 and March 2010. Data for a second cohort, all women who had cytology, was available from an extract of the national screening database (Exeter system, taken in October/November 2010), including monthly attendances to the screening programme from April 2007 to March 2010.^3^ This resulted in a census of cytology results. Information in this extract included the women’s age in 5-year groups, category of invitation (eg. routine recall (3 or 5 years after a negative screen, depending on age), early recall after an abnormality, surveillance after treatment) and test result. All cervical cytology was read using British Society for Clinical Cytology (BSCC) terminology in laboratories subject to national accreditation and quality assurance. A comparison between the British and the Bethesda terminology is given by the National Cancer Screening Service.^4^

Data from the National Audit of Invasive Cervical Cancers in England was used for the results of cytology in women with cervical cancer.^5–7^ We excluded all follow-up tests. For women with cervical cancer we defined the ‘index test’ as the first test within 12 months of diagnosis. This time period was chosen to allow time for diagnosis following early (6-month) recall triggered by low-grade cytology, whilst trying to ensure that the cancer was already present at the time of cytology. In both women with cancer and in the general population, cytology taken due to an earlier abnormal result (recall cytology) was excluded.

We considered two groups of women in whom cytology was outside of routine call and recall for cervical screening: those who had cytology taken age ≥70 (‘post-screening’), who were not on follow up for a previous abnormal result; and women who had their first cytology test at age 40–69 (‘late prevalent test’). Routine screening in England ceases at a woman’s 65^th^ birthday, but women invited at 64 may be screened at 65, and women with abnormal screening tests (or colposcopy) in their 60s may continue to have cervical cytology in their mid 60s. It is therefore likely that the majority of tests in women aged 65–69 would be related to the screening programme, rather than being symptomatic. By contrast virtually all cytology taken aged ≥70 would not be related to a screening invitation.

Additionally, we compare the age-adjusted stage distribution of a third cohort of women, those diagnosed with cervical cancer aged 66 and over (ie. after routine screening ceases in England) between April 2007 and March 2012, by whether they had cytology within 12 months of diagnosis. In this group, the date range of diagnosis was extended, to include more cancers in this cohort. Stage was not recorded for 24% of these women. Two methods of assigning stage to those with unknown stage were considered. The first used the stage distribution for all women with known stage; the second did this separately for those with and without cytology in the year before diagnosis. This was combined with 5-year relative survival data from SEER (Surveillance, Epidemiology, and End Results^8^) to estimate the reduction in case fatality that could be achieved if all women of this age had cytology prior to their index test. SEER data from the USA were used, as survival data by stage and age are not readily available for England. For comparative purposes, we also used English stage-specific survival data, which are not available by age.^9^ Finally, we compared the stage distribution across age groups in women aged ≥40 who did not have cytology in the 12 months before diagnosis.

The positive predictive values (PPVs) were calculated by dividing the number of cancers diagnosed with a given index test result by the number of cytology tests during the same period with that same test result. The number of women needed to test (NNT) is the number of cytology tests divided by the number of cancers diagnosed following moderate or worse cytology. The 95% confidence intervals (CI) for the NNT and the PPV were calculated assuming that the number of cancers diagnosed from the number of cytology tests carried out had a binomial distribution.^10^ Analyses were carried out in STATA 12 (StataCorp. College Station, Texas, USA).

## Results

Between 1 April 2007 and 31 March 2010 93,322 cytology tests were conducted in the cohort of women whose first cytology test was at age 40–69 (late prevalent test), and 14,668 for women aged ≥70 (post-screening). There were 259 women diagnosed with cervical cancer within a year of their first cytology aged 40–69, and 78 women aged ≥70, who also had a cytology test within 12 months of diagnosis, during the same period. The prevalence of cancer among late prevalent test women was 2.8 per 1000, and 5.3 per 1000 for post-screening women. In the census of cytology results, 2.5% of the tests were high grade (moderate or worse, equivalent to high grade cervical squamous intraepithelial lesion (HSIL)+) for post-screening women, including 2.3% severe or worse (Table 1a), and the corresponding numbers were 1.4% and 1.1% in late prevalent test women (Table 1b). In post-screening women 89.1% of tests were negative (Table 1a) and 90.8% were negative in late prevalent test women (Table 1b). In post-screening women 5.5% of tests were inadequate (Table 1a); 3.6% were inadequate in late prevalent test women (Table 1b). The NNT to identify one cancer (based on referring women with a result of moderate or worse) in late prevalent test women was 404 (95% CI: 355–462) and 226 (95% CI: 177–292) in post-screening women.

**Table 1.** Result of the first (non-recall) cytology test in the last 12 months in women (a) aged ≥70 and (b) whose first cytology test was at age 40–69, with cervical cancer (‘Cancers') and in the general population (‘Cytology tests') and predictive value (PV) of the test result to cervical cancer.

	a) Aged ≥70					b) First cytology aged 40–69				
Cytology test result^1^	Cancers	% cancers diagnosed with test result or worse	Cytology tests	% of all tests	PV	Cancers	% cancers diagnosed with test result or worse	Cytology tests	% of all tests	PV
Negative	4	100	13071	89.1	0.03%	2	100	84,774	90.8	0.00%
Inadequate	2	95	809	5.5	0.25%	6	99	3,381	3.6	0.18%
Borderline	7*	92	350	2.4	2.00%	14*	97	2,554	2.7	0.55%
Mild	0	83	74	0.5	0.00%	6	92	1,275	1.4	0.47%
Moderate	3	83	21	0.1	14.29%	12	89	311	0.3	3.86%
Severe	8	79	47	0.3	17.02%	108	85	705	0.8	15.32%
?Glandular	11	69	184	1.3	5.98%	10	43	128	0.1	7.81%
?Invasive	43	55	112	0.8	38.39%	101	39	194	0.2	52.06%
**Total**	**78**		**14668**		**0.53%**	**259**		**93322**		**0.28%**
Moderate or worse	65	83	364	2.5	17.86%	231	89	1338	1.4	17.26%

1 First routine cytology test in the last 12 months.

* 5/7 and 9/14 were referred to colposcopy based on a single borderline, suggesting that they may have been labelled “query high grade, equivalent to ASC-H”.

**Figure 1.**
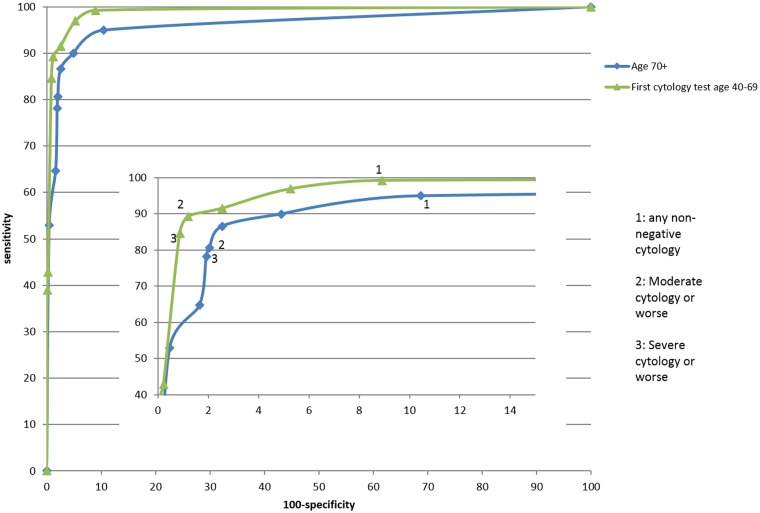
ROC curves showing the sensitivity and specificity of each cytology result for cervical cancer (i) in women aged 70+ and (ii) in women whose first cytology was at age 40–69.

The sensitivity of moderate or worse cytology for cancer was 83% (95% CI: 73%–91%) in post-screening women and 89% (95% CI: 85%–91%) in late prevalent test women. The PPV for cervical cancer of a query invasive report was over 35% in both groups (Table 1). The PPV of a report of query glandular neoplasia was between 6% and 8%, and for a report of severe dyskaryosis was between 15% and 17% (Table 1). The PPV of moderate dyskaryosis was much higher for post-screening women than for late prevalent test women (13.0% vs. 3.9%, p = 0.026). The receiver operating characteristic (ROC) curves are shown in Figure 1, with an area under the curve of 96.2% (95% CI: 93.5%–98.9%) for post-screening women and 98.7% (95% CI: 98.1%–99.3%) for late prevalent test women.

Table 2 shows the stage distribution of women diagnosed with cervical cancer aged ≥66, by whether they had cytology in the 12 months before diagnosis, excluding any women who had an abnormal result on their previous cytology test. Women with cytology in the 12 months prior to diagnosis were diagnosed at an earlier stage than women without. If the stage distribution in women without cytology were to be changed to the distribution in women with cytology, their case fatality could be reduced by 17.3%–26.4% (depending on assumptions regarding the stage distribution in those with unknown stage, Table 2). The stage distribution for women aged ≥40 without cytology in the 12 months before diagnosis is largely independent of age (result not shown).

**Table 2.** Five-year age-adjusted case fatality rates by stage at diagnosis in those aged ≥66 diagnosed April 2007-March 2012 and the possible reduction if the stage distribution of women with cytology in the 12 months prior to diagnosis was achieved.

Stage	1A	1B	2	3	4	not recorded	Total	Age-adjusted case fatality (scenario 1)^2^	Age-adjusted case fatality (scenario 2)^3^	Crude case fatality^4^
Case fatality (%) SEER^1a^	1.6	16.2	47.4	67.5	89.8					
Case fatality (%) Anglia^1b^	4	4	46	62	95					
women with cytology within 12 months of diagnosis aged ≥66										
N	9	44	41	13	14	34	155*	42.0%	39.7%	35.0%
%	5.8	28.4	26.5	8.4	9.0	21.9				
women without cytology within 12 months of diagnosis aged ≥66										
N	49	285	373	275	235	398	1615	50.7%	51.0%	47.6%
%	3.0	17.6	23.1	17.0	14.6	24.6				
Relative reduction in case fatality (%)								17.3	22.1	26.4

1a 100 minus 5-year % relative survival for women aged 70+, SEER 1988–2001 (see reference 15).

1b 100 minus 5-year % relative survival (all ages), Anglia 2002–2006 (http://www.cancerresearchuk.org/health-professional/cancer-statistics/statistics-by-cancer-type/cervical-cancer/survival#heading-Three).

2 Assuming the stage distribution for those with stage not recorded did not depend on having cytology in the year prior to diagnosis and using SEER survival.

3 Assuming the stage distribution for those with stage not recorded did depend on having cytology in the year prior to diagnosis and using SEER survival.

4 Ignoring those with unrecorded stage and using survival from Anglia.

* 78 of these cancers were part of the cohort aged ≥70 and 10 of the cohort diagnosed within a year of their first cytology test aged 40–69 in Table 1.

## Discussion

We have shown elsewhere that the PPV of cytology for invasive cervical cancer using a cut-off of severe or worse cytology is 5.4% in a screening population (aged 20–64).^2^ The PPV (even at moderate or worse cytology) is even higher among women aged 40–69 who have not been previously screened (PPV 21.3%) and women aged ≥70 (PPV 18.1%). The sensitivity of cytology with a cut-off of moderate dyskaryosis or worse for invasive cancer is 89% and 83% respectively, in these high-risk populations, and levels of inadequate results were low. Thus cytology is an excellent differentiator between cancer and non-cancer among women tested outside routine call and recall for cervical screening. Furthermore, we show an earlier stage at diagnosis among women aged ≥66 with cytology within 12 months of diagnosis compared with those with no such cytology, corresponding to a 17–26% reduction in case fatality. In these high-risk populations, the numbers of cancers diagnosed per 1000 women tested (2.8 per 1000 in women with their first test aged 40–69 and 5.3 per 1000 for women aged ≥70) are comparable with those identified by bowel screening (2.0 per 1000 for faecal occult blood testing)^11^ and mammographic screening (8.1 per 1000).^12^

The main strength of this study is that cytology results were available for the entire population of England, with data on time since previous screen, which allowed women with no previous screening results to be identified, together with the linked screening histories for women diagnosed with cervical cancer.

The main limitation is the lack of information on the reason for the cytology tests. In particular we have no data on symptoms. However, because this publication demonstrates high sensitivity and excellent PPV of cytology for invasive cancer outside of routine screening, it is important to publicize the results to stimulate further studies looking at symptoms specifically. It is not possible to rule out reverse causation as a (partial) explanation for the better stage distribution observed in women with cytology within a year of diagnosis, for example, if some women with advanced cancer first present with severe symptoms that lead to diagnosis without cytology. This study reports cytology read using BSCC terminology in laboratories subject to accreditation and quality assurance, and the conclusions may not be generalizable to other systems for reporting cytology, or to countries with less quality assurance. Survival data for England are not available by both stage and age, and survival for stages 1A and 1B are combined. Because the proportion of stage 1 cancers that are stage 1A falls off rapidly with age, using the average stage 1 survival grossly overestimates the survival of women with stage 1 cancer in this study, most of whom had stage 1B cancer. This led to an overestimation of the reduction in case fatality associated with cytology in the 12 months before diagnosis. Although survival rates in the USA are slightly better than in England, this is true for all stages of diagnosis, and should therefore not unduly affect the estimated relative reduction in case fatality.

We have studied cytology in women aged ≥70 and in women whose first cytology was at age 40–69. The cytology in these women was unlikely to have been purely for screening. Based on high rates of prevalent cervical cancer, we infer that many of these cytology tests are likely to have been in women with symptoms (why else would a woman in her 70s have cytology in England?). An audit of cervical cancers diagnosed in a London hospital showed that women with ‘symptomatic’ cytology tests were less likely to have early stage cancer than screen-detected cancers in asymptomatic women, though more likely than women without cytology.^13^

Diagnosing symptomatic cervical cancer in primary care is challenging, given that gynaecological symptoms are common in women with non-malignant conditions (eg. genital infections), but cervical cancer is relatively rare. This study suggests that cervical cytology could be an important triage tool for primary care physicians managing women with gynaecological symptoms. Cervical cytology in this context should be considered as an additional test to be used alongside the normal investigations and management of non-specific gynaecological symptoms (especially genital infections), and should not be treated in the same way as screening cytology. We propose that GPs could be responsible for arranging cytology samples in women with symptoms who attend an appointment (preferably to be taken by an experienced smear taker, at that appointment). Samples collected from symptomatic women not due for screening should be specially labelled.

The reason cited for not recommending cytology in symptomatic women is that it may delay diagnosis either directly, through waiting unnecessarily for the test result, or, more worryingly, as the result of the false reassurance provided by a false-negative test.^14^ However, we found that women with non-screening cytology prior to diagnosis are diagnosed at earlier stages than women without such cytology. There is no evidence that cytology delayed the diagnosis of cancer in these women. It is also widely thought that both cytology and colposcopy are problematic in older women. We have shown here that cytology is excellent for detecting invasive cancers in older women, and it appears that older women with abnormal smears are diagnosed with earlier staged cancers, which is presumably happening through colposcopy. Older women could be given a short course of oestrogen prior to colposcopy, as is often done currently. If cytology were introduced as a triage test for early identification of cervical cancer in women with symptoms, it would seem rational to ignore all results reported as less than high grade, unless the woman was (over)due for screening. Women with a negative, borderline or mild dyskaryosis result would be managed as if no cytology had been done. Our proposed strategy for referral is shown in Box 1. In practice we suggest that ASC-H, which is rare, should be included with HSIL, as there is evidence that a small, but not insignificant, proportion of cancers are in women with ASC-H.^2^ This strategy would result in 2–3% of symptomatic women being referred to colposcopy, a tiny addition to the current workload in England. As shown here, even a negative cytology does not rule out cancer, and in patients with unremitting symptoms, referral to a gynaecologist would still be appropriate.

**Box 1.** Proposed management of symptomatic women based on cytology result.

BSCC cytology result	Bethesda System result	Proposed management
Negative	Within normal limits	Continue to explore cause of symptoms. Do not rule out cervical cancer.
Inadequate*	Unsatisfactory*	
Borderline dyskaryosis*^	ASC-US*	
Mild dyskaryosis*	LSIL*	
Moderate dyskaryosis	HSIL	Urgent referral to colposcopy
Severe dyskaryosis	HSIL
Query glandular neoplasia	AIS	Urgent (2 week) referral to colposcopy. Consider differential diagnosis of endometrial cancer in post-menopausal women with query glandular neoplasia on cytology
Query squamous cell carcinoma	Query invasive	

* If the woman was overdue screening, test for HPV DNA on residual sample and refer for colposcopy if HPV positive. ^Borderline test results where high grade disease cannot be excluded and where no HPV test is reported should be considered for referral to colposcopy.

## Conclusion

This study suggests that cervical cytology at a threshold of HSIL in women being tested outside of routine call and recall for cervical screening is an excellent identifier of cervical cancer, and could facilitate earlier diagnosis. We believe that the evidence presented warrants evaluation of cytology as a triage tool in women presenting to primary care with unexplained gynaecological symptoms.

## References

[1] WHO. World Health Organisation national cancer control programmes. http://www.who.int/cancer/nccp/en/ 10th July 2014.

[2] Landy R, Castanon A, Hamilton W, Lim AWW, Dudding N, Hollingworth A, Sasieni PD. Evaluating cytology for the detection of invasive cervical cancer. *Cytopathology*. Online first: 30 Jun 2015. DOI: 10.1111/cyt.12259. 10.1111/cyt.12259 PMC4913744 26126636

[3] Lancucki L Sasieni P Patnick J Day T Vessey M The impact of Jade Goody's diagnosis and death on the NHS Cervical Screening Programme. Journal of Medical Screening 2012; 19(2): 89–93. 22653575 10.1258/jms.2012.012028 PMC3385661

[4] National Cancer Screening Service. Guidelines for Quality Assurance in Cervical Screening. 2009.

[5] Sasieni P, Castanon A, Louie K. NHSCSP Audit of Invasive Cervical Cancer. National Report 2007–2010. 2011.

[6] Sasieni P, Castanon A, Cuzick J. Effectiveness of cervical screening with age: population based case-control study of prospectively recorded data. *BMJ: British Medical Journal* 2009;339. 10.1136/bmj.b2968 PMC2718082 19638651

[7] The NHS Information Centre Screening and Immunisations team. Cervical Screening Programme - England, 2010–11: Report. 2011.

[8] Kosary CL. SEER Survival Monograph: Cancer Survival Among Adults: U.S. SEER Program, 1988–2001, Patient and Tumor Characteristics, Chapter 14. National Cancer Institute, SEER Program, NIH Pub. No. 07-6215. Bethesda, MD: 2007.

[9] Cancer Research UK. Cervical cancer survival by stage at diagnosis 2014 [cited 2015 16th June 2015]. Available from: http://www.cancerresearchuk.org/health-professional/cancer-statistics/statistics-by-cancer-type/cervical-cancer/survival#heading-Three.

[10] Altman DG Confidence intervals for the number needed to treat. BMJ: British Medical Journal 1998; 317(7168): 1309–1309. 9804726 10.1136/bmj.317.7168.1309 PMC1114210

[11] Cancer Research UK. About bowel cancer screening 2014 [updated 12th November 2014; cited 2014 28th November]. Available from: http://www.cancerresearchuk.org/about-cancer/type/bowel-cancer/about/screening/about-bowel-cancer-screening.

[12] Health and Social Care Information Centre - Screening and Immunisations team. Breast Screening Programme, England 2011–12. 2013.

[13] Herbert A Culora G Dunsmore H Gupta S Holdsworth G Kubba A Invasive cervical cancer audit: why cancers developed in a highâ risk population with an organised screening programme. BJOG: An International Journal of Obstetrics & Gynaecology 2010; 117(6): 736–45. 20184570 10.1111/j.1471-0528.2010.02511.x

[14] McCartney M. Doctors and patients confuse cervical screening with diagnostic tests. *BMJ: British Medical Journal* 2014;348. 10.1136/bmj.g3334 24846386

